# Mid-life outcomes of young people’s antisocial behavior: the role of developmental heterogeneity across childhood and adolescence

**DOI:** 10.1017/S0033291725000789

**Published:** 2025-04-28

**Authors:** Gurleen Popli, Barbara Maughan, Richard Rowe

**Affiliations:** 1 School of Economics, University of Sheffield, Sheffield, UK; 2 Social, Genetic and Developmental Psychiatry Centre, Institute of Psychiatry, Psychology and Neuroscience, King’s College London, London, UK; 3 School of Psychology, University of Sheffield, Sheffield, UK

**Keywords:** BCS70, conduct problems, developmental pathways, Childhood and adolescence Social, health, and economic outcomes

## Abstract

**Background:**

Antisocial behavior (ASB) is relatively common in childhood and adolescence. While it harms victims, perpetrators are at increased risk of disadvantageous adult outcomes. Developmental heterogeneity is well documented; distinctions have been drawn between early-onset persistent, adolescent-onset, and childhood-limited pathways. We examine whether individuals in some pathways face worse mid-life outcomes than others and whether the pattern differs across sexes.

**Methods:**

The 1970 British Cohort Study assessed parent-reported ASB measures at ages 5, 10, and 16. We classified developmental pathways using the Rutter A scale conduct questions. We categorized children scoring in the top 10% of the distribution as showing high ASB, separately at each assessment. Approximately 6000 individuals were classified into low (73%), childhood-limited (11%), adolescent-onset (9%), and early-onset persistent (7%) groups. We tested associations of ASB grouping with age 46 social, economic, and health outcomes, controlling for a range of covariates.

**Results:**

The childhood-limited group showed little mid-life difficulty. The early-onset persistent and adolescent-onset groups both showed a pattern of worse midlife outcomes for boys and girls.

**Conclusions:**

The results highlight that ASB in young people is not transient and that prevention and treatment during early childhood and adolescence are warranted.

## Key points

- Childhood and adolescent antisocial behavior can follow different developmental trajectories. Little is known about mid-life outcomes of different antisocial pathways particularly for females.
- We examined psychosocial outcomes in the 1970 British Cohort Study at age 46.
- Individuals on adolescent-onset and early-onset persistent trajectories faced increased risk of many disadvantageous psychosocial outcomes, including arrest and smoking, while childhood-limited antisocial behavior was not strongly associated with disadvantageous mid-life outcomes.
- Results showed some sex-specific patterns: in males, ASB was associated with caution and convictions and number of partners, while in females it was more associated with psychological distress and lower mental well-being.
- These results highlight the importance of reducing antisocial behavior in young people to reduce disadvantageous long-term outcomes for individuals and for society.

## Introduction

Antisocial behavior (ASB) in young people is costly for victims and perpetrators. Young people who engage in ASB are at increased risk of a broad range of disadvantageous outcomes in adulthood spanning physical health (e.g., overweight, accelerated aging), mental health (e.g., internalizing problems, substance use), and psychosocial (e.g., criminal conviction, social isolation) domains (Erskine et al., 2016; Wertz et al., 2018). This, in turn, leads to involvement with health, justice, and welfare services, aggregating to substantial societal costs (Rissanen et al., 2021; Rivenbark et al., 2018). Developmental heterogeneity in ASB is long recognized. Individuals following varied trajectories during childhood and adolescence may face differing adult outcomes. Identifying those at elevated risk for disadvantageous outcomes can inform prevention and treatment priorities.

Moffitt’s (1993) developmental taxonomy has been influential in research and clinical practice regarding ASB heterogeneity, as illustrated by its influence on subtyping conduct disorder in DSM-IV (American Psychiatric Association, 1994) and DSM5 (American Psychiatric Association, 2013). The taxonomy distinguished life-course persistent ASB, which onsets during early childhood and persists into adulthood from adolescent-limited ASB, where disruptiveness begins following puberty and declines when adulthood begins. Subsequent research has identified an additional childhood-limited pathway, where ASB onsets during childhood and desists during adolescence. The developmental taxonomy model hypothesized that risk factors for trajectories are qualitatively distinct: life-course persistent ASB was posited to be linked to early individual characteristics, such as hyperactivity and neuropsychological deficits, and early family characteristics, such as ineffective parenting and low socioeconomic status (Moffitt, 1993). These risk factors were not hypothesized to be involved in adolescence-limited ASB, which was instead linked to biological maturity occurring earlier than social maturity. In this ‘maturity gap’ ASB is hypothesized to facilitate the rewards and status demanded by biological maturity but unavailable due to social immaturity. Moffitt (2018) reviews evidence supporting this position concerning ASB in boys. Fairchild, van Goozen, Calder, and Goodyer (2013) proposed a revised taxonomic model in which risk factors for ASB onset at different ages vary quantitatively rather than qualitatively, with earlier onset associated with more severe risk exposure. Evidence consistent with this position has been reported from several epidemiological studies (Jolliffe, Farrington, Piquero, Loeber, & Hill, 2017; Martins-Silva et al., 2024).

In terms of adult outcomes, Moffitt’s developmental taxonomy predicted that ASB would continue into adulthood for those on a life-course persistent trajectory. It was hypothesized that those following an adolescent-limited trajectory would have more desistance opportunities in adulthood (Moffitt, 2018). It was noted, however, that the consequences of adolescent ASB may have implications, such as impaired educational achievement and incarceration, that can trap individuals in antisocial lifestyles. Therefore, some individuals on an adolescence-limited pathway may continue their ASB during adulthood. On Fairchild et al.’s (2013) revised taxonomy, the positive correlation between ASB severity and earlier onset may imply the poorest adult outcomes in those that start earliest, with lower levels of impact in those with later onsets.

A growing literature has assessed the adult functioning of individuals following different ASB trajectories. In describing these studies and our analyses, we label developmental trajectories during childhood and adolescence independently from adult outcomes. We refer to ASB that begins in childhood and persists to adolescence as early-onset persistent (EOP). We describe ASB that begins during adolescence as adolescent-onset (AO) and maintain the childhood-limited (CL) label for ASB that onsets during childhood and offsets in adolescence. The early adult outcomes of different developmental ASB trajectories were assessed in a meta-analysis of 13 studies (including 10,633 participants, mean age 22.5 years, maximum 32 years; Bevilacqua, Hale, Barker, & Viner, 2018). EOP children were at high risk of disadvantageous outcomes across psychosocial (criminality, lower educational, and employment levels), mental health (depressive symptomatology, alcohol use), and general physical health domains. The AO group was also at elevated risk of disadvantageous outcomes, although their risk was often lower than in the EOP group. The authors noted that the measurement of outcomes early in adulthood may have emphasized negative outcomes in the AO group, if they were following an adolescent-limited trajectory, as the offset of their ASB would have had limited opportunity to translate into normative psychosocial outcomes. The CL group had poorer outcomes than those who did not display ASB as young people, but the effects were generally less marked than for other ASB groups.

Since the meta-analysis was published, outcomes at ages 38 and 45 have been examined in the Dunedin study. ASB developmental trajectories formed using data collected between ages 7 and 26 (Rivenbark et al., 2018) found that the EOP group used more criminal justice, health, and social welfare services than other groups. The AO group was elevated on most service uses but generally to a lesser extent than the EOP group. The CL group used more services than those following a stable-low ASB trajectory. Langevin et al. (2022) found the EOP group displayed more advanced signs of biological aging at age 45 than individuals following other trajectories. Both the AO and CL groups also showed accelerated aging but less so than the EOP group. Differences between groups were explained by adjusting for a range of covariates, including self-control difficulties in early life and smoking.

Further work is required to expand our understanding of these issues. First, most reviewed studies cover young adult outcomes; less is known about mid-life outcomes linked with ASB developmental trajectory. Second, boys have more commonly been studied than girls and application to girls is unclear (Freitag et al., 2018). Odgers et al. (2008) found similar developmental trajectories in girls and boys in the Dunedin study, although EOP was less common in girls than boys. In this study, the pattern of outcomes at age 32 showed many similarities between girls and boys across measures of violence, physical health, mental health, and economic problems. Other authors have proposed that AO in girls may be associated with characteristics more typical of the EOP pathways in boys (Konrad et al., 2021; Silverthorn & Frick, 1999), including impaired adult outcomes (Fontaine, Carbonneau, Vitaro, Barker, & Tremblay, 2009). In addition, outcomes of ASB in girls may differ from boys, for example, with differing impacts of unplanned parenthood. Therefore, it is important to explore adult outcomes of different ASB trajectories in girls and boys separately.

Our analyses address these knowledge gaps. We examine a range of mid-life (age 46) outcomes for boys and girls following EOP, AO, and CL ASB developmental patterns through childhood and adolescence in the 1970 British Cohort Study (BCS70). BCS70 includes a large community sample followed from birth to middle age, with ASB measured at ages 5, 10, and 16. To match the wide range of outcomes studied in previous literature (Rivenbark et al., 2018), we include a range of variables spanning the psychosocial, physical, and mental health domains and showing sufficient variation at age 46 to allow meaningful analysis. Previous analyses have demonstrated the suitability of BCS70 for our purpose; childhood conduct problems have been shown to predict criminal convictions (Murray, Irving, Farrington, Colman, & Bloxsom, 2010), life satisfaction (Layard, Clark, Cornaglia, Powdthavee, & Vernoit, 2014), and educational qualifications and social class at ages 30–34 (Lewis, 2018). We build on this work to identify the age 46 social, economic, and health outcomes for boys and girls following different antisocial trajectories through childhood and adolescence. We examine the impact of covariates, including social disadvantage, hyperactivity, intellectual functioning, and academic achievement. These analyses test whether the effects of the ASB pathway are independent from correlates of childhood and adolescent disruptive behavior that may also increase risk of difficulties in adulthood.

## Method

### Participants

BCS70 follows all children born in England, Scotland, and Wales during 1 week in April 1970 (Elliott & Shepherd, 2006). The ‘birth wave’ had a sample of 17,196 Cohort Members (CMs). Of these, 8,978 CMs provided data at ages 5, 10, and 16 years. Among these 8,978, parents of 6,645 CMs provided a complete conduct problems history, supporting ASB pathway formation. After taking into account family characteristics data, we have complete information on 5,909 CMs. There were 8,581 CMs with productive age 46 interviews, but data availability varied across outcomes. Appendix S1 (Supporting Information) details how the data were collected at each wave, and the varying observations numbers across adult outcomes. Given BCS70 attrition, we applied inverse probability weights (see Appendix S1).

### Measures

#### Antisocial behavior

At ages 5, 10, and 16, parents (usually the mother) completed the Rutter A Scale (Rutter, Tizard, & Whitmore, 1970). We used questions addressing conduct: (i) often destroys own or others’ belongings; (ii) sometimes takes things belonging to others; (iii) is often disobedient; (iv) often tells lies; and (v) bullies other children. At ages 5 and 16, the response categories were ‘does not apply’ (coded 0), ‘applies somewhat’ (1), and ‘certainly applies’ (2). We summed responses to provide a continuous score (range 0–10). At age 10, responses used a visual analogue scale; parents were shown a horizontal line labelled 0 (does not apply) to 100 (certainly applies), which they bisected with a vertical line. The line drawn was reported as a number 0–100 and was summed across the five items (range 0–500). We categorized children scoring in the top 10% of the distribution as showing high ASB, separately at ages 5, 10, and 16. This provides similar cutoffs to one standard deviation above the sample mean, a threshold used elsewhere (Moffitt, Caspi, Dickson, Silva, & Stanton, 1996).

Previous research on ASB developmental heterogeneity has often employed growth mixture modelling (Nguena Nguefack et al., 2020) to identify trajectory groups, including analyses of the BCS70 Rutter scale data (Girard & Okolikj, 2024). However, with three data points (ages 5, 10, and 16), this approach is inherently limited to fitting linear trajectories. This does not fit all the hypothesized trajectories; the CL group are hypothesized to display high ASB during childhood (i.e., at 5 and/or 10) and low ASB at age 16. The AO group are hypothesized to have low ASB at both ages 5 and 10 and high ASB at age 16. These trajectories are not naturally modelled by linear terms, and we note that Girard and Okolikj (2024) did not find an AO trajectory. To form groups matching, the hypothesized developmental pathways we did not use trajectory modelling but instead classified trajectories based on the pattern of ASB scores across development. No ASB: ASB was not high at ages 5, 10, or 16; CL: high ASB was reported at 5 and/or 10, but not 16; AO: high ASB was reported at age 16 only; and EOP: high ASB was reported at 5 and/or 10 and 16.

#### Adult outcomes

Adult outcomes were assessed at age 46, excluding cautions and convictions (age 34).

*Cautions and conviction:* A binary indicator coded 1 if the CM has responded yes to either *‘have you ever been found guilty by a court*’, or *‘have you ever been formally cautioned at the police station’* since adolescence at either age 30 or 34; and 0 otherwise. This question was not asked after age 34.

*Social and economic outcomes:* (1) Currently lives with a partner was measured as a binary indicator endorsed if the CM is currently living with a partner/spouse; (2) Number of partners, was generated using the CM’s partnership history from age 26 onward (Hancock & Peters, 2021). We define a categorical variable taking value 0 (never reported having a partner), 1 (has ever had one partner), 2 (two partners), and 3 (3 or more partners). The maximum number of partners reported is 9. (3) The CM’s economic outcome is captured by a binary indicator, high skill job, taking value 1 if their current job was ‘higher managerial and administrative’, the National Statistics Socio-Economic Classification (National Statistics, 2014) scheme’s highest category, and 0 otherwise; this is not captured for those who have never worked or are long-term unemployed.

*General health:* (1) Self-assessed health was reported on a Likert scale (Jylhä, 2009). We defined a binary indicator scoring 1, where health was rated excellent or very good, and 0 for good, fair or poor; (2) Disability, defined as a substantial or long-term negative effect on daily activities, was a binary variable based on three impairment questions using a BCS70 algorithm (www.bcs70.info).

*Mental health:* (1) Psychological distress was measured using the Malaise Inventory (Rutter et al., 1970), which provides a count score (range 0–9), with higher scores indicating greater difficulties; (2) life satisfaction was measured on a 10-point Likert scale from completely dissatisfied to completely satisfied, converted to a standardized continuous scale (mean = 0, sd = 1); (3) well-being was measured using the Warwick-Edinburgh mental well-being scale (Tennant et al., 2007), which contains 14 items scored 1–5 that form a continuous scale, standardized (mean = 0 and sd = 1) with higher scores indicating better well-being.

*Physical health:* (1) Body mass index, was treated as a continuous variable. (2) Dominant hand grip strength was measured using the Smedley spring-gauge dynamometer in kilograms and treated as a continuous score. Weaker grip strength indexes risk for health problems, including cardiovascular disease, respiratory disease, and cancer (Celis-Morales et al., 2018).

*Health behaviours:* Thigh-worn activPAL3 micro devices (PAL Technologies Ltd., Glasgow, UK) were worn by 6,492 participants and 5,569 provided usable data. These measured (1) mean daily hours of moderate to vigorous activity, and (2) daily step count for 7 days. (3) Problematic alcohol consumption was measured using the Alcohol Use Disorders Identification Test – Primary Care (Babor, Higgins-Biddle, Saunders, & Monteiro, 2001), which contains 5 questions scored 0–4. Problematic alcohol consumption was defined as scoring >4. (4) Current smoking was a binary indicator based on self-report.

#### Covariates

We included birthweight, ethnicity (captured by a white/non-white binary indicator), and age 10 Rutter A scale hyperactivity score. Intellectual functioning was included as average reading score over the age 5 Schonell Reading Test, and the Shortened Edinburgh Reading Test measured at ages 10 and 16 (Parsons, 2014). We also included family socioeconomic status at birth as measured by mothers’ age at CM’s birth, a binary indicator capturing whether the mother had education beyond age 15, and a binary indicator indexing whether the father had a managerial, technical, or professional occupation. We included a binary indicator to capture family stability, scored 1 if the parents were together throughout the CM’s childhood; and 0 if not (Wood, Stafford, & O’Neill, 2019). Maternal psychological distress was indicated when mother had a high Malaise Inventory score when the CM was aged 5, 10, or 16 (Hancock & Johnson, 2013). Lastly, to check robustness, we used highest completed education qualification at age 30 as an additional covariate. Educational qualifications were captured by three binary indicators of whether the CM has achieved GCSE/O-levels, A-levels, or a degree or above; base category is no qualifications.

### Analyses

Analyses were conducted using Stata 18 (StataCorp, 2023) including sampling weights to minimize non-random attrition biases. Observations were weighted to the inverse of their sampling probability (see Appendix S1). We first estimated the association between ASB group and outcomes without covariates. Next, we re-estimated models including covariates. OLS regressions were used to analyze continuous outcomes and logit models, reporting odds ratios, were used for binary outcomes. We estimated a negative binomial model and reported incidence rate ratios when predicting the Malaise count measure. For number of partners, an ordered logit model was estimated. Models were parametrized to compare each ASB trajectory to the no ASB group. We estimated models separately for boys and girls.

## Results

ASB was more common in boys; 32% were in one of the ‘high’ ASB categories compared to 22% of girls. The AO group was similarly frequent in boys (9.5%) and girls (8.8%), but boys fell more commonly into the CL (13.3% vs 8.6%) and EOP (9.0% vs 4.7%) groups. Table S2.1 (Supporting Information, Appendix S2) shows the ASB groups were more exposed to most childhood risks (treated as covariates) than the non-ASB group, for girls and boys. The EOP group was at greater risk than the no ASB group on all covariates in boys and all in girls except for low birth weight and maternal age. Compared to other ASB groups, the EOP group was most at risk on many covariates including father’s occupational status, maternal malaise, family instability, reading score, and hyperactivity across boys and girls. Statistical comparisons between groups (Table S2.1, Supporting Information, Appendix S2) show that the EOP group had significantly lower reading scores and higher hyperactivity than the other ASB groups in boys and girls. The EOP group also had significantly worse maternal malaise scores than the AO group in boys but not girls. No other covariates varied significantly between EOP and AO groups. The AO group showed substantial levels of childhood adversity with significantly elevated risk on all covariates except ethnic-minority status in boys and birth weight and maternal age in girls. The AO group was often at lower risk than the EOP group although comparisons were rarely significant, with some exceptions noted above. The CL group showed significantly elevated risk relative to no ASB group on most covariates except for maternal age (boys and girls) and birth weight in boys. Relative to other ASB groups, the CL group was at lowest risk on some childhood measures, for example family stability and maternal age. Other covariates showed a different pattern, however. In boys and girls, the CL group showed significantly higher hyperactivity than the AO group although lower than the EOP group. In boys, reading scores were significantly lower in the CL group than the AO group but these groups were not significantly different in girls.

Table S2.3 (Supporting Information, Appendix S2) shows educational achievements were significantly lower in all ASB groups than in the non-ASB group for boys and girls except for the CL group for girls. After including covariates, the CL and non-antisocial group were not significantly different in boys as well. The EOP and AO groups remained significantly impaired on educational outcomes independently of covariates. The 95% confidence intervals show that coefficients for AO and EOP groups did not differ statistically.

Tables 1A and 1B report unadjusted descriptive statistics for the outcomes by ASB pathway, for males and females, respectively. Generally, the ASB groups faced more difficulties during mid-life than the non-ASB group. Table 2 reports group comparisons for males and females after covariate adjustment. Table S2.4 (Supporting Information, Appendix S2) shows unadjusted results.

**Table 1A.** Descriptive statistics for outcomes in MALES: mean for continuous variables and proportions for dichotomous variables (number of observations in parentheses)

	No ASB	Childhood limited	Adolescent onset	Early onset persistent	Total
Caution or conviction^a^	0.28 (1633)	0.41 (294)	0.48 (212)	0.59 (189)	0.35 (2328)
***Social and economic outcomes***					
Lives with a partner^a^	0.77 (1090)	0.88 (189)	0.82 (129)	0.71 (100)	0.79 (1508)
Number of partners^b^	1.4 (1090)	1.5 (189)	1.6 (129)	1.7 (100)	1.5 (1508)
High skill job^a^^,^^c^	0.3 (1090)	0.23 (189)	0.094 (129)	0.086 (100)	0.25 (1508)
***General health***					
Self-assessed health^a^	0.57 (944)	0.52 (171)	0.46 (115)	0.34 (89)	0.53 (1319)
Disability^a^	0.1 (944)	0.13 (171)	0.25 (115)	0.13 (89)	0.13 (1319)
***Mental health***					
Psychological distress (Malaise count)	1.4 (944)	1.5 (171)	1.5 (115)	1.6 (89)	1.4 (1319)
Life satisfaction	0.002 (944)	0.021 (171)	−0.4 (115)	−0.22 (89)	−0.06 (1319)
Warwick Edinburgh mental well-being scale	0.054 (944)	−0.089 (171)	−0.36 (115)	−0.042 (89)	−0.019 (1319)
***Physical health***					
BMI	29 (944)	29 (171)	29 (115)	30 (89)	29 (1319)
Grip strength dominant hand (kilograms)	45 (944)	46 (171)	49 (115)	49 (89)	46 (1319)
***Health behaviors***					
Problematic alcohol consumption^a^	0.32 (1180)	0.27 (208)	0.25 (151)	0.39 (119)	0.31 (1658)
Smoking current^a^	0.19 (1180)	0.27 (208)	0.34 (151)	0.53 (119)	0.25 (1658)
Daily step counts	9755 (742)	9779 (138)	8507 (82)	8309 (58)	9503 (1020)
Mean activity (minutes/day)	52 (742)	52 (138)	42 (82)	41 (58)	50 (1020)

a Dichotomous variables

b Number of partners variable takes four values: 0 (never reported having a partner), 1 (has ever had one partner), 2 (has ever had two partners), and 3 (has ever had 3 or more partners). The maximum number of partners any CM reports is 9.

c Among those who are employed.

The descriptive statistics have been weighted, using Inverse Probability Weights (IPW) calculated by the authors. See Appendix S1 for details on how IPWs were obtained.

**Table 1B.** Descriptive statistics for outcomes in FEMALES: mean for continuous variables and proportions for dichotomous variables (number of observations in parentheses)

	No ASB	Childhood limited	Adolescent onset	Early onset persistent	Total
Caution or conviction^a^	0.061 (2110)	0.061 (214)	0.072 (221)	0.13 (116)	0.067 (2661)
***Social and economic outcomes***					
Lives with a partner^a^	0.74 (1332)	0.62 (129)	0.59 (116)	0.59 (65)	0.7 (1642)
Number of partners^b^	1.5 (1332)	1.5 (129)	1.4 (116)	1.6 (65)	1.5 (1642)
High skill job^a^^,^^c^	0.12 (1332)	0.085 (129)	0.15 (116)	0.021 (65)	0.12 (1642)
***General health***					
Self-assessed health^a^	0.56 (1314)	0.57 (114)	0.37 (116)	0.28 (60)	0.53 (1604)
Disability^a^	0.2 (1314)	0.21 (114)	0.57 (116)	0.45 (60)	0.25 (1604)
***Mental health***					
Psychological distress (Malaise count)	1.8 (1314)	2 (114)	3.1 (116)	2.9 (60)	2 (1604)
Life satisfaction	0.15 (1314)	−0.24 (114)	−0.17 (116)	−0.46 (60)	0.043 (1604)
Warwick Edinburgh mental well-being scale	0.1 (1314)	−0.14 (114)	−0.79 (116)	−0.59 (60)	−0.058 (1604)
***Physical health***					
BMI	28 (1314)	28 (114)	29 (116)	30 (60)	29 (1604)
Grip strength dominant hand (kilograms)	27 (1314)	27 (114)	27 (116)	27 (60)	27 (1604)
***Health behaviors***					
Problematic alcohol consumption^a^	0.15 (1613)	0.19 (158)	0.17 (153)	0.17 (83)	0.16 (2007)
Smoking current^a^	0.18 (1613)	0.2 (158)	0.33 (153)	0.35 (83)	0.21 (2007)
Daily step counts	8959 (1024)	8340 (88)	8716 (98)	7959 (42)	8820 (1252)
Mean activity (minutes/day)	49 (1024)	47 (88)	47 (98)	42 (42)	48 (1252)

a Dichotomous variables

b Number of partners variable takes four values: 0 (never reported having a partner), 1 (has ever had one partner), 2 (has ever had two partners), and 3 (has ever had 3 or more partners). The maximum number of partners any CM reports is 9.

c Among those who are employed.

The descriptive statistics have been weighted, using Inverse Probability Weights (IPW) calculated by the authors. See Appendix S1 for details on how IPWs were obtained.

**Table 2.** Contrast between childhood antisocial behavior groups and adult outcomes: For binary outcomes odds ratios (OR) from a logit model, and for continuous outcomes estimated coefficients from an OLS regression are reported

	Males			Females		
	CL versus No ASB	AO versus No ASB	EOP versus No ASB	CL versus No ASB	AO versus No ASB	EOP versus No ASB
Caution or conviction (OR)	1.39 [0.85, 2.25]	2.08** [1.20, 3.63]	3.24*** [2.03, 5.17]	0.69 [0.23, 2.06]	0.97 [0.39, 2.39]	1.81 [0.70, 4.70]
***Social and economic outcomes***						
Lives with a partner (OR)	2.45** [1.26, 4.80]	1.39 [0.68, 2.84]	0.89 [0.41, 1.96]	0.73 [0.32, 1.66]	0.8 [0.37, 1.72]	0.66 [0.27, 1.64]
Number of partners (OR)^a^	1.78** [1.19, 2.65]	1.8 [0.95, 3.42]	2.33* [1.16, 4.65]	0.83 [0.40, 1.72]	0.83 [0.37, 1.88]	0.96 [0.52, 1.77]
High skill job (OR)^b^	1.07 [0.56, 2.03]	0.25*** [0.12, 0.53]	0.35** [0.16, 0.77]	1 [0.35, 2.91]	1.84 [0.71, 4.75]	0.24* [0.06, 0.95]
***General health***						
Self-assessed health (OR)	1.03 [0.64, 1.67]	0.68 [0.35, 1.32]	0.51 [0.26, 1.00]	1.62 [0.72, 3.63]	0.72 [0.37, 1.40]	0.46* [0.22, 0.97]
Disability (OR)	1.18 [0.59, 2.36]	3.06** [1.34, 6.99]	1.14 [0.39, 3.31]	0.73 [0.30, 1.74]	3.82*** [1.92, 7.59]	2.33 [0.99, 5.50]
***Mental health***						
Malaise count (IRR)	1 [0.74, 1.34]	1.05 [0.73, 1.52]	1.06 [0.66, 1.71]	0.83 [0.57, 1.21]	1.47* [1.04, 2.07]	1.21 [0.83, 1.76]
Life satisfaction	0.16 [−0.03, 0.34]	−0.31* [−0.59, −0.03]	−0.02 [−0.38, 0.33]	−0.15 [−0.47, 0.17]	−0.15 [−0.50, 0.20]	−0.42 [−0.93, 0.09]
WE mental well-being scale	−0.03 [−0.19, 0.14]	−0.34 [−0.76, 0.08]	0.05 [−0.30, 0.41]	0.08 [−0.18, 0.34]	−0.64** [−1.13, −0.16]	−0.45 [−1.22, 0.32]
***Physical health***						
BMI	0.02 [−1.00, 1.04]	0.35 [−0.68, 1.39]	0.8 [−0.43, 2.03]	−0.6 [−2.44, 1.24]	−0.43 [−2.17, 1.31]	1.3 [−1.61, 4.22]
Grip strength dominant hand	1.01 [−1.07, 3.10]	3.53 [−0.04, 7.10]	4.31** [1.47, 7.15]	−0.14 [−1.92, 1.65]	0.43 [−1.51, 2.37]	0.71 [−1.49, 2.91]
***Health behaviors***						
Problematic alcohol consumption (OR)	0.87 [0.52, 1.44]	0.68 [0.38, 1.25]	1.34 [0.68, 2.63]	1.01 [0.47, 2.16]	1.09 [0.53, 2.26]	0.95 [0.36, 2.52]
Smoking current (OR)	1.06 [0.57, 1.95]	2.01* [1.10, 3.67]	3.49*** [1.78, 6.83]	0.94 [0.44, 1.99]	2.08* [1.07, 4.04]	1.32 [0.53, 3.28]
Daily step counts	22.72 [−881.05, 926.50]	−1346.29 [−2792.42, 99.85]	−1558.48 [−3665.87, 548.90]	−327.98 [−1308.64, 652.68]	104.72 [−1196.14, 1405.57]	−882.69 [−2579.82, 814.44]
Mean activity (minutes/day)	−0.3 [−6.66, 6.07]	−9.58 [−19.66, 0.50]	−11.15 [−23.45, 1.16]	0.22 [−6.79, 7.24]	1.52 [−7.51, 10.55]	−4.59 [−15.66, 6.47]

*Notes:* [.] are 95% confidence intervals. *p < 0.05, **p < 0.01, ***p < 0.001

a Odds ratios from an ordered logit model

b Among those who are employed

CL , Childhood limited; AO , Adolescent onset; EOP , Early onset persistent; IRR , Incidence rate ratio

All regressions adjust for covariates: Birthweight; ethnicity (captured by a white/non-white binary indicator); mother’s age at birth of the CM; a binary indicator if mother had education beyond age 15; a binary indicator if father had a managerial, technical or professional occupation; age 10 Rutter A scale hyperactivity score; average reading score over the ages 5, 10 and 16; a binary indicator to capture family stability, 1 if the parents were together throughout the CM’s childhood; and 0 if the parents divorced by the time the CM was 16 years old; a binary indicator for maternal psychological distress, if mother had a high Malaise Inventory score when the CM was aged 5, 10 or 16.

IPWs are used in all regressions.

Table 2 shows age 34 cautions and convictions were elevated in the EOP and AO groups relative to the no ASB group for males. Cautions and convictions were less common in the CL group than in other ASB groups and did not differ from the No ASB group. In females, cautions and convictions were not associated with ASB grouping.

*Social and economic outcomes*: *Living with a partner* and *number of partners* only significantly varied across ASB groups in males. Males in the CL group were more likely to be living with a partner than those in the no ASB group. Males in the CL and EOP groups were likely to have had more partners compared to the no ASB group. In males, the AO and EOP were less likely to have *held a high skilled job.* In females, the EOP group that was less likely to have held a high skill job than the no ASB group.

*General health: Self-assessed health* was significantly lower in the EOP group relative to the No ASB group for females. Self-assessed health was unrelated to ASB in males, although the comparison between EOP and No ASB was only just above p < .05. *Disability status* was significantly more common in the AO group relative to No ASB for males and females.

*Mental health and wellbeing:* Psychological distress, as measured by the *Malaise Inventory*, was elevated in the AO group in females but in no other groups. Well-being, as measured by *life satisfaction*, was lower only in the AO group for males and was unrelated to antisocial group in females. In contrast, the *Warwick-Edinburgh Well-being Scale* was lower in the AO group for females but unrelated to ASB group in males.

*Physical health:* ASB groupings were unrelated to *BMI* in both sexes. The EOP group had stronger *grip strength* than the non-antisocial group among men and these variables were unrelated in women.

*Health behaviors: Problematic alcohol consumption* was unrelated to ASB group in both sexes. *Current smoking* was more common in the AO and EOP groups in men and the AO group in women. Physical activity, as measured by *daily step count* and *time spent physically active*, was unrelated to ASB group status in men or women.

## Discussion

We examined mid-life outcomes of people following different ASB pathways during childhood and adolescence. We contribute to the literature by measuring a wide range of outcomes well into mid-life using a large sample containing males and females. BCS70 provides a strong dataset for our purpose, including a large nationally representative sample measuring ASB at appropriate developmental stages and assessing relevant midlife outcomes. Despite these strengths, the results must be considered in the context of some limitations. First, we employed a classification scheme based on ASB levels at each measurement point, rather than latent class growth curve modelling. We discussed our rationale in the Measures section. We identified trajectory groupings of comparable size to those found in latent class analyses (e.g., Rivenbark et al., 2018) and we observed a compatible pattern of correlates during childhood and adolescence and outcomes in adulthood. Second, despite controlling for numerous covariates, the influence of unmeasured factors cannot be entirely ruled out.

We found the antisocial groups experienced significantly more disadvantageous mid-life outcomes compared to the no ASB group, including caution and conviction and many social, economic, and health difficulties. Analyses conducted without covariates (Appendix S2, Table S2.4) provide results very similar to those with covariates. We explored whether lower educational achievement, observed in the AO and EOP groups, mediated the link between adolescent ASB and disadvantageous outcomes. One hypothesis is that adolescent ASB disrupts educational outcomes, which, in turn, increases risk of difficulties in adulthood. We found educational achievements were lower in AO and EOP groups. However, re-analyzing our data controlling for educational achievements (available on request) revealed that controlling for education minimally impacted the results, suggesting that education did not fully mediate the effects of adolescent ASB.

We focus on group differences that were independent from covariates and therefore more likely to represent the independent outcomes of ASB. There was some sex variation in the pattern of mid-life outcomes. For example, male ASB was related to caution or conviction and having had more relationships, whereas female ASB was not. Current smoking was elevated in AO and EOP males while it was only elevated in AO females. While these results might indicate EOP males may be more prone to future physical illness, EOP males specifically showed stronger grip strength than those with no ASB, which is typically associated with *lower* risk of cardiovascular and respiratory diseases, and cancer. Conversely, female ASB was more strongly linked to higher psychological distress and lower mental well-being.

In males, the EOP group was most impaired in outcomes, including caution and conviction, number of partners, and current smoking. There were also outcomes where the AO group was more disadvantaged than the EOP group, including holding a high skilled job, having a disability, and lower life satisfaction. In most cases, the confidence intervals on the coefficients indicated that risk did not differ significantly between the EOP and AO groups. This may reflect a lack of statistical power to distinguish risk between groups (as the number of observations in some categories is small, as seen in Tables 1A and 1B).

The AO and EOP groups showed a pattern of disadvantaged outcomes in females too. EOP females were less likely to achieve a highly skilled job or report self-assessed good health. However, a greater number of disadvantageous outcomes were significantly impacted in the AO group, including disability, psychological distress, mental well-being, and current smoking. As in males, inspection of the confidence intervals indicates few comparisons between the EOP and AO groups are statistically significant. These results are consistent with evidence that females starting ASB during adolescence may be heterogeneous. It has been suggested that, in addition to some girls following the adolescence-limited trajectory, other girls that onset in adolescence may follow an adolescence-delayed-onset trajectory with many characteristics similar to the male EOP pathway (Fontaine et al., 2009; Silverthorn & Frick, 1999).

Overall, these findings are consistent with other studies that show individuals following an AO trajectory suffer substantial adult difficulties. This was evident in Bevilacqua et al.’s (2018) review of early adult outcomes. Our results indicate that the difficulties of those in the AO group extend into mid-life, as Langevin et al. (2022) found regarding aging markers. Our results regarding mid-life outcomes may be interpreted using Moffitt’s (2018) developmental taxonomy or the revised developmental taxonomy model proposed by Fairchild et al. (2013), which proposes differences between EOP and AO individuals are quantitative rather than qualitative. We note that the AO group displayed markers of elevated risk during childhood relative to those in the No ASB group, for example, in terms of hyperactivity, while often showing lower levels of these factors than the EOP group. This pattern fits with Fairchild et al.’s (2013) model and is compatible with results from other cohort studies (Jolliffe et al., 2017; Martins-Silva et al., 2024).

In contrast, the CL group showed little mid-life impairment. In males, members of the CL group had a higher number of partners but were also more likely to be co-habiting currently. We did not find impaired outcomes in the female CL group. These findings suggest less adult difficulties in the CL group than reported elsewhere; the Bevilacqua et al. (2018) review concluded that the CL group showed less impairment than the other ASB trajectories but demonstrated poorer educational and aggressive outcomes than those without ASB. Our finding may reflect that ASB confined to childhood has diminishing effects as adulthood progresses.

## Conclusion

Our findings support the importance of treatment and prevention of ASB during early childhood, given the poor outcomes observed for the EOP group. Parenting programs offer one well-documented intervention route (Leijten, Melendez-Torres, & Gardner, 2021). In addition, targeting childhood risk factors that are elevated in the EOP pathway may be valuable for prevention. Our comparison of childhood difficulties faced by the EOP and CL groups may offer pointers to such intervention targets. Among other risks, EOP children were more likely to have high levels of hyperactivity, lower reading ability, and more family instability than CL children. These difficulties may be addressed by medical (Storebø et al., 2015), educational (Machin, McNally, & Meghir, 2004), and family instability (Loft, 2020) interventions, respectively. Targeting such factors may help to divert children displaying ASB during childhood down the CL route, which our findings show is associated with fewer difficulties in adulthood. Our findings also highlight the importance of reducing conduct problems during adolescence; our AO group suffered a range of disadvantageous outcomes, often at similar levels to the EOP group. While multisystemic therapy has been recommended to treat ASB during adolescence (McCart & Sheidow, 2016), more recent evaluations provide less promising evidence of universal efficacy (Littell, Pigott, Nilsen, Green, & Montgomery, 2021). Identifying effective adolescent treatments may be particularly important for reducing the burden of ASB given the similarity of outcomes for the AO and EOP groups in our analyses.

## Supporting information

Popli et al. supplementary material Popli et al. supplementary material

## Supplementary material

The supplementary material for this article can be found at http://doi.org/10.1017/S0033291725000789.
