# Supplementary material for: Mid-life outcomes of young people’s antisocial behavior: the role of developmental heterogeneity across childhood and adolescence
Source: Psychol Med. 2025 Apr 28;55:e124. doi: 10.1017/S0033291725000789 (PMC12094618; doi:10.1017/S0033291725000789)
Supplement: Popli et al. supplementary material [file S0033291725000789sup001.docx]

**Supporting Information**

**Mid-life outcomes of young people’s antisocial behaviour: The role of developmental heterogeneity across childhood and adolescence**

Gurleen Popli^a^, Barbara Maughan^b^ & Richard Rowe^c^

^a.^ School of Economics, University of Sheffield, Sheffield, UK

^b.^ Social, Genetic and Developmental Psychiatry Centre, Institute of Psychiatry, Psychology and Neuroscience, King’s College London, London, UK

^c.^ Department of Psychology, University of Sheffield, Sheffield, UK

**January 2025**

**Appendix S1: Sample attrition, inverse probability weights, and data collection**

The 1970 British Birth Cohort Study (BCS70) follows a community sample born in England, Scotland, and Wales during one week in April 1970. Data at birth (wave 1) were collected using a questionnaire completed by the midwife who was present at the birth and, in addition, information was extracted from clinical records. In the subsequent two waves, at ages 5 (wave 2) and 10 (wave 3), data was collected from the cohort members (CMs), their parents, and their schools; at home interviews were conducted by child health visitors. Age 16 (wave 4) information was collected from CMs, their parents, and schools, via separate youth, parental and school questionnaires, respectively. Subsequently, information was collected from CMs only. At age 46 a full set of bio-measures were administered by a nurse. See Elliott & Shepherd (2006) for further details of the data collected.

The ‘birth wave’ had a sample of 17,196 CMs whose families provided a productive interview. A productive interview does not mean all questions were answered, just that enough were answered to be included in the specific wave. Of the 17,196 families which were productive in the birth wave, only 8,978 CMs had productive interviews in the first four waves (i.e. at birth, and ages 5, 10 and 16 years). There were 8,581 productive interviews at age 46. The biggest reason for attrition is individuals moving to a new address and not being subsequently traced, refusal rates (once contacted) were low. Plewis et al. (2004) provide a detailed analysis of attrition in BCS70.^[[1]](#footnote-1)^ Table S1.1 details the survey response.

**Table S1.1: 1970 British Cohort Study, Survey Response**

| Wave | Productive interviews at each wave | Productive interviews for wave 1 – 4* |
| --- | --- | --- |
| BCS1 (Birth) | 17,196 | 17,196 |
| BCS2 (Age 5)^+^ | 13,135 (76.4%) | 12,748 (74.1%) |
| BCS3 (Age 10) | 14,875 (86.5%) | 11,810 (68.7%) |
| BCS4 (Age 16) | 11,615 (67.5%) | 8,978 (52.2%) |
| BCS10 (Age 46) | 8,581 (49.9%) |  |

* Productive interviews in a given wave, including those present for all previous waves.

^+^ There were some CMs who should have been included in the BCS70 study based on its original sample design, but were missed in the birth wave, these were then included in the study later, which could be at wave 2, 3 or 4. For these CMs there is no birth wave data. This explains the difference in the row numbers for BCS2.

(.) give the response rate from the birth wave.

Among those who had productive interviews in the first four waves, for 6,645 CMs we have a complete history of conduct problem measures allowing us to create the antisocial behaviour (ASB) pathways; however, we lose further observations because of non-response on the other covariates used to capture individual and family characteristics, this leaves complete information on 5,909 CMs. We match the outcomes from the age 46 sample for these 5,909 CMs from the first four waves; this gives us a different number of observations for each outcome, as not every CM provided information on all outcomes.

Given the attrition, we use inverse probability weights in our analysis. We have 15 outcomes at age 46, we divide our outcomes into five groups to define the number of complete cases. These groupings with the number of complete case observations are given in Table S1.2. For each set of outcomes, we have the dependent variable $R_{i}$, for the logit regression, taking value 1 for the complete cases, and 0 for the rest of the individuals. The predictors used in the logit regression are from the birth wave. These include covariates used in the main analysis (birth weight, binary variable for non-white, mother’s age at birth of CM, binary variable for mother’s education beyond age 15, binary variable for father having high occupation at birth of the CM), and an additional set of covariates (binary variable for mother married at birth of CM, binary variable if breastfeeding was attempted at birth of the CM, parity, binary variable for father’s education beyond age 15, a binary variable indicating if father’s education information is missing, father’s age at birth of CM, a binary variable if father’s age is missing, binary variable for sex of the CM). Table S1.3 gives us the estimates from the logit regression for each of the five sets of outcomes. In total we will have 17,185 observations, this is the sample from the birth wave. Table S1.4 gives the descriptive statistics for the weights.

**Table S1.2: Complete cases at age 46**

|  | Outcome | Complete cases |
| --- | --- | --- |
| (1) | Caution & Conviction | 4,989 |
| (2) | Social and economic outcomes: lives with a partner, number of partners, and high-skill jobs | 3,150 |
| (3) | General health: self-assessed health, disability status;  Mental health: Malaise score, life satisfaction, Warwick Edinburg scale; and  Physical health: BMI, grip strength. | 2,923 |
| (4) | Health behaviours: problematic alcohol consumption, smoking current, daily step count. | 3,665 |
| (5) | Health behaviours: physical activity | 2,272 |

**Table S1.3: Logit regressions for IPWs, where the dependent variable takes value 1 for the complete cases, and 0 for the remaining individuals.**

|  | (1) | (2) | (3) | (4) | (5) |
| --- | --- | --- | --- | --- | --- |
|  | Caution & conviction | Socio-Economic outcomes | Health (general, mental, physical) | Health behaviours (excluding physical activity) | Physical activity |
| Birth weight | 1.00*** | 1.00*** | 1.00*** | 1.00*** | 1.00*** |
|  | [1.00,1.00] | [1.00,1.00] | [1.00,1.00] | [1.00,1.00] | [1.00,1.00] |
| Non-white | 0.49*** | 0.47*** | 0.33*** | 0.44*** | 0.35*** |
|  | [0.38,0.63] | [0.34,0.65] | [0.23,0.48] | [0.32,0.59] | [0.23,0.53] |
| Mother's age at birth of CM | 1.02*** | 1.02*** | 1.02*** | 1.02*** | 1.02** |
|  | [1.01,1.03] | [1.01,1.03] | [1.01,1.03] | [1.01,1.03] | [1.01,1.03] |
| Mother education 15+ | 1.23*** | 1.29*** | 1.34*** | 1.29*** | 1.28*** |
|  | [1.13,1.34] | [1.17,1.42] | [1.21,1.48] | [1.18,1.41] | [1.15,1.42] |
| Father high occupation, at birth | 1.05 | 1.10 | 1.12 | 1.10 | 1.07 |
|  | [0.95,1.16] | [0.98,1.23] | [1.00,1.25] | [0.99,1.22] | [0.94,1.21] |
| Mother married | 1.15 | 1.01 | 1.05 | 0.98 | 0.89 |
|  | [0.95,1.40] | [0.81,1.26] | [0.83,1.32] | [0.79,1.20] | [0.70,1.14] |
| Breastfeeding attempted | 1.07 | 1.14** | 1.17*** | 1.16*** | 1.14** |
|  | [0.99,1.15] | [1.04,1.24] | [1.07,1.28] | [1.07,1.26] | [1.03,1.25] |
| Parity | 0.87*** | 0.85*** | 0.86*** | 0.85*** | 0.85*** |
|  | [0.84,0.90] | [0.82,0.88] | [0.82,0.90] | [0.81,0.88] | [0.81,0.89] |
| Father education 15+ | 1.08 | 1.12* | 1.16** | 1.11* | 1.13* |
|  | [0.99,1.18] | [1.01,1.23] | [1.05,1.29] | [1.01,1.22] | [1.01,1.26] |
| Father education missing | 0.95 | 0.86 | 0.91 | 0.86 | 0.90 |
|  | [0.77,1.19] | [0.66,1.12] | [0.69,1.19] | [0.68,1.10] | [0.67,1.21] |
| Father's age at birth of CM | 0.99 | 1.00 | 0.99 | 1.00 | 1.00 |
|  | [0.99,1.00] | [0.99,1.01] | [0.99,1.00] | [0.99,1.01] | [0.99,1.01] |
| Father's age missing | 0.06*** | 0.08*** | 0.08*** | 0.08*** | 0.08*** |
|  | [0.05,0.08] | [0.06,0.10] | [0.06,0.11] | [0.06,0.11] | [0.06,0.12] |
| Male | 0.70*** | 0.79*** | 0.69*** | 0.67*** | 0.69*** |
|  | [0.65,0.75] | [0.73,0.86] | [0.63,0.75] | [0.62,0.73] | [0.63,0.76] |
| *N* | 17185 | 17185 | 17185 | 17185 | 17185 |

*Notes*: Exponentiated coefficients; 95% confidence intervals in brackets.

* p<0.05, ** p<0.01, *** p<0.001

**Table S1.4: Descriptive statistics for the weights**

|  | (1) | (2) | (3) | (4) | (5) |
| --- | --- | --- | --- | --- | --- |
|  | Caution & conviction | Socio-Economic outcomes | Health (general, mental, physical) | Health behaviours (excluding physical activity) | Physical activity |
| **Dependent variable = 0** | | |  |  |  |
| Mean | 12.01 | 18.78 | 20.30 | 16.07 | 27.13 |
| Median | 3.21 | 5.06 | 5.83 | 4.60 | 7.23 |
| SD | 13.18 | 23.06 | 26.94 | 19.98 | 37.49 |
| Min | 1.68 | 2.12 | 2.13 | 1.86 | 2.59 |
| Max | 165.61 | 377.10 | 405.50 | 339.01 | 627.47 |
| P25 | 2.47 | 3.86 | 4.16 | 3.32 | 5.23 |
| P75 | 20.93 | 33.35 | 33.57 | 26.96 | 45.16 |
| *Count* | *12196* | *14035* | *1426* | *13520* | *14913* |
| **Dependent variable = 1** | | |  |  |  |
| Mean | 3.51 | 5.59 | 5.94 | 4.79 | 7.58 |
| Median | 2.42 | 3.83 | 4.06 | 3.25 | 5.15 |
| SD | 5.14 | 8.79 | 8.58 | 7.37 | 11.34 |
| Min | 1.56 | 2.04 | 1.99 | 1.78 | 2.71 |
| Max | 130.71 | 178.45 | 110.22 | 175.80 | 155.94 |
| P25 | 2.16 | 3.23 | 3.32 | 2.76 | 4.31 |
| P75 | 2.83 | 4.59 | 5.14 | 4.02 | 6.54 |
| *Count* | *4989* | *3150* | *2923* | *3665* | *2272* |
| **Total** |  |  |  |  |  |
| Mean | 9.54 | 16.36 | 17.86 | 13.66 | 24.55 |
| Median | 2.84 | 4.68 | 5.33 | 4.12 | 6.75 |
| SD | 12.07 | 21.79 | 25.38 | 18.63 | 35.79 |
| Min | 1.56 | 2.04 | 1.99 | 1.78 | 2.59 |
| Max | 165.61 | 377.10 | 405.50 | 339.01 | 627.47 |
| P25 | 2.33 | 3.68 | 3.95 | 3.14 | 5.03 |
| P75 | 17.09 | 28.33 | 28.24 | 22.37 | 40.25 |
| *Count* | *17185* | *17185* | *17185* | *17185* | *17185* |

*Notes:* P25 is the 25^th^ percentile and P75 is the 75^th^ percentile.

**Appendix S2: Tables**

**Table S2.1: Descriptive statistics of covariates (Mean and Standard deviation) by categories of ASB**

|  | **Male** | | | | **Female** | | | |
| --- | --- | --- | --- | --- | --- | --- | --- | --- |
| **Covariates till age 16 of CM** | **No ASB** | **CL** | **AO** | **EOP** | **No ASB** | **CL** | **AO** | **EOP** |
| Birth weight (grams) | 3417.86 | 3374.17 | 3309.98 | 3275.63 | 3284.72 | 3204.67 | 3230.51 | 3245.68 |
|  | (528.29) | (569.07) | (590.15) | (554.59) | (503.62) | (452.09) | (526.56) | (555.32) |
| Non-white ^a^ | 0.01 | 0.03 | 0.01 | 0.03 | 0.01 | 0.04 | 0.05 | 0.04 |
|  | (0.11) | (0.17) | (0.12) | (0.17) | (0.12) | (0.19) | (0.21) | (0.20) |
| Mother’s age at birth of CM | 26.37 | 25.94 | 25.29 | 24.43 | 26.12 | 25.73 | 25.52 | 24.80 |
|  | (5.16) | (5.70) | (5.47) | (5.41) | (5.22) | (5.24) | (5.65) | (5.63) |
| Mother education 15+ ^a^ | 0.41 | 0.29 | 0.30 | 0.26 | 0.39 | 0.32 | 0.27 | 0.31 |
|  | (0.49) | (0.45) | (0.46) | (0.44) | (0.49) | (0.47) | (0.45) | (0.47) |
| Father high occupation, at birth of CM ^a^ | 0.22 | 0.15 | 0.14 | 0.10 | 0.22 | 0.13 | 0.14 | 0.10 |
|  | (0.42) | (0.36) | (0.35) | (0.30) | (0.41) | (0.34) | (0.35) | (0.30) |
| Mother Malaise ^a^ | 0.25 | 0.42 | 0.41 | 0.54 | 0.26 | 0.51 | 0.48 | 0.56 |
|  | (0.43) | (0.49) | (0.49) | (0.50) | (0.44) | (0.50) | (0.50) | (0.50) |
| Family stable ^a^ | 0.84 | 0.77 | 0.70 | 0.67 | 0.83 | 0.77 | 0.65 | 0.59 |
|  | (0.37) | (0.42) | (0.46) | (0.47) | (0.38) | (0.42) | (0.48) | (0.49) |
| Average reading score | 0.27 | -0.16 | 0.02 | -0.34 | 0.22 | -0.10 | -0.13 | -0.32 |
|  | (0.90) | (0.96) | (0.98) | (1.04) | (0.90) | (1.00) | (0.96) | (1.02) |
| Hyperactivity score, age 10 | -0.12 | 0.40 | 0.15 | 0.87 | -0.33 | 0.28 | 0.04 | 0.52 |
|  | (0.89) | (1.05) | (1.02) | (1.12) | (0.78) | (1.07) | (1.01) | (1.18) |

*Notes:*  ^a^ Dichotomous variables, for these variables the reported descriptive statistics is their proportions.

CL = Childhood limited, AO = Adolescent onset, EOP = Early onset persistent.

**Table S2.2: P-values for the test of covariate means across ASB groups**

|  | **CL vs.**  **No ASB** | **AO vs.**  **No ASB** | **EOP vs.**  **No ASB** | **AO vs.**  **CL** | **EOP vs.**  **CL** | **EOP vs.**  **AO** |
| --- | --- | --- | --- | --- | --- | --- |
| **Panel A: Male** |  |  |  |  |  |  |
| Birth weight (grams) | 0.150 | **0.002** | **<0.001** | 0.136 | **0.024** | 0.467 |
| Non-white ^a^ | **0.010** | 0.814 | **0.033** | 0.104 | 0.972 | 0.147 |
| Mother’s age at birth of CM | 0.148 | **0.002** | **<0.001** | 0.123 | **<0.001** | 0.062 |
| Mother education 15+ ^a^ | **<0.001** | **<0.001** | **<0.001** | 0.843 | 0.429 | 0.361 |
| Father high occupation, at birth of CM ^a^ | **0.003** | **0.003** | **<0.001** | 0.726 | 0.070 | 0.173 |
| Mother Malaise ^a^ | **<0.001** | **<0.001** | **<0.001** | 0.720 | **0.001** | **0.001** |
| Family stable ^a^ | **0.003** | **<0.001** | **<0.001** | **0.033** | **0.002** | 0.357 |
| Average reading score | **<0.001** | **<0.001** | **<0.001** | **0.017** | **0.017** | **<0.001** |
| Hyperactivity score, age 10 | **<0.001** | **<0.001** | **<0.001** | **0.001** | **<0.001** | **<0.001** |
| **Panel B: Female** |  |  |  |  |  |  |
| Birth weight (grams) | **0.015** | 0.095 | 0.368 | 0.555 | 0.434 | 0.771 |
| Non-white ^a^ | **0.010** | **<0.001** | **0.024** | 0.417 | 0.805 | 0.665 |
| Mother’s age at birth of CM | 0.261 | 0.078 | **0.004** | 0.645 | 0.090 | 0.188 |
| Mother education 15+ ^a^ | **0.018** | **<0.001** | 0.061 | 0.265 | 0.947 | 0.385 |
| Father high occupation, at birth of CM ^a^ | **0.001** | **0.002** | **<0.001** | 0.920 | 0.381 | 0.335 |
| Mother Malaise ^a^ | **<0.001** | **<0.001** | **<0.001** | 0.390 | 0.288 | 0.074 |
| Family stable ^a^ | **0.019** | **<0.001** | **<0.001** | **0.001** | **<0.001** | 0.173 |
| Average reading score | **<0.001** | **<0.001** | **<0.001** | 0.703 | **0.020** | **0.044** |
| Hyperactivity score, age 10 | **<0.001** | **<0.001** | **<0.001** | **0.001** | **0.008** | **<0.001** |

*Notes:* CL = Childhood limited, AO = Adolescent onset, EOP = Early onset persistent.

Bold indicates significance at or below 5% level.

**Table S2.3: Educational qualifications: descriptive statistics and odds ratios from logit model**

|  | **Proportions**  **Educational qualification** | | | | **Odds ratios**  **Dependent variable: less than degree educational qualification** | |
| --- | --- | --- | --- | --- | --- | --- |
|  | **None** | **GCSE/O-levels** | **A levels** | **Degree or above** | **No covariates** | **Covariates included** |
| **Male** |  |  |  |  |  |  |
| No ASB | 0.25 | 0.34 | 0.07 | 0.34 | 1.00 | 1.00 |
|  |  |  |  |  | - | - |
| Childhood limited | 0.30 | 0.40 | 0.06 | 0.24 | 1.65*** | 1.08 |
|  |  |  |  |  | [1.24, 2.20] | [0.78, 1.49] |
| Adolescent onset | 0.37 | 0.42 | 0.04 | 0.17 | 2.45*** | 1.94*** |
|  |  |  |  |  | [1.70, 3.53] | [1.32, 2.84] |
| Early onset persistent | 0.51 | 0.37 | 0.04 | 0.08 | 5.80*** | 3.59*** |
|  |  |  |  |  | [3.44, 9.76] | [2.03, 6.35] |
| **Female** |  |  |  |  |  |  |
| No ASB | 0.20 | 0.38 | 0.09 | 0.33 | 1.00 | 1.00 |
|  |  |  |  |  | - | - |
| Childhood limited | 0.30 | 0.40 | 0.03 | 0.27 | 1.36 | 0.96 |
|  |  |  |  |  | [1.00, 1.86] | [0.68, 1.36] |
| Adolescent onset | 0.33 | 0.46 | 0.08 | 0.14 | 3.16*** | 2.24*** |
|  |  |  |  |  | [2.14, 4.66] | [1.48, 3.41] |
| Early onset persistent | 0.43 | 0.38 | 0.06 | 0.13 | 3.20*** | 1.89* |
|  |  |  |  |  | [1.87, 5.46] | [1.05, 3.39] |

*Notes*: [.] give the 95% confidence intervals for the odds ratios. * p<0.05, ** p<0.01, *** p<0.001

Dependent variable takes 1 if the CM had less than degree, and 0 = degree or above education.

**Table S2.4:** **Contrast between childhood antisocial behaviour groups and age 46 outcomes: For binary outcomes odds ratios (OR) from a logit model, and for continuous outcomes estimated coefficients from an OLS regression are reported. No adjustment for covariates.**

|  | **Males** | | | **Females** | | |
| --- | --- | --- | --- | --- | --- | --- |
| **Dependent variables:** | **CL vs. No ASB** | **AO vs. No ASB** | **EOP vs. No ASB** | **CL vs. No ASB** | **AO vs. No ASB** | **EOP vs. No ASB** |
|  |  |  |  |  |  |  |
| Caution or conviction (OR) | 1.79 | 2.44** | 3.79*** | 0.99 | 1.18 | 2.32 |
|  | [0.95, 3.37] | [1.36, 4.34] | [2.37, 6.05] | [0.37, 2.68] | [0.54, 2.62] | [0.96, 5.60] |
| ***Social and economic outcomes*** |  |  |  |  |  |  |
| Lives with a partner (OR) | 2.16* | 1.33 | 0.71 | 0.58 | 0.51 | 0.51 |
|  | [1.19, 3.92] | [0.64, 2.76] | [0.33, 1.54] | [0.25, 1.34] | [0.22, 1.18] | [0.21, 1.25] |
| Number of partners (OR) ^a^ | 1.46 | 1.79 | 2.35** | 0.86 | 0.76 | 1.22 |
|  | [0.85, 2.54] | [0.82, 3.91] | [1.27, 4.34] | [0.40, 1.82] | [0.33, 1.71] | [0.69, 2.14] |
| High skill job (OR) ^b^ | 0.69 | 0.24*** | 0.22*** | 0.67 | 1.25 | 0.16** |
|  | [0.37, 1.29] | [0.13, 0.46] | [0.11, 0.43] | [0.22, 1.99] | [0.40, 3.95] | [0.05, 0.53] |
| ***General health*** |  |  |  |  |  |  |
| Self-assessed health (OR) | 0.79 | 0.64 | 0.38** | 1.02 | 0.45* | 0.30** |
|  | [0.50, 1.25] | [0.31, 1.33] | [0.19, 0.76] | [0.48, 2.17] | [0.22, 0.91] | [0.13, 0.67] |
| Disability (OR) | 1.33 | 2.85* | 1.35 | 1.08 | 5.33*** | 3.32** |
|  | [0.68, 2.61] | [1.14, 7.12] | [0.51, 3.59] | [0.46, 2.52] | [2.51, 11.34] | [1.36, 8.14] |
| ***Mental health*** |  |  |  |  |  |  |
| Malaise count (IRR) | 1.12 | 1.11 | 1.19 | 1.13 | 1.75** | 1.64* |
|  | [0.81, 1.55] | [0.77, 1.60] | [0.78, 1.83] | [0.73, 1.75] | [1.21, 2.51] | [1.11, 2.44] |
| Life satisfaction | 0.02 | -0.41* | -0.22 | -0.38* | -0.32 | -0.60* |
|  | [-0.14, 0.18] | [-0.72, -0.09] | [-0.59, 0.15] | [-0.76, -0.01] | [-0.71, 0.07] | [-1.14, -0.06] |
| WE mental well-being scale | -0.14 | -0.41 | -0.1 | -0.24 | -0.89** | -0.69 |
|  | [-0.29, 0.01] | [-0.87, 0.04] | [-0.45, 0.26] | [-0.50, 0.02] | [-1.42, -0.36] | [-1.57, 0.19] |
| ***Physical health*** |  |  |  |  |  |  |
| BMI | 0.05 | 0.43 | 1.01 | -0.03 | 0.12 | 1.86 |
|  | [-0.97, 1.07] | [-0.54, 1.40] | [-0.13, 2.15] | [-1.86, 1.79] | [-1.42, 1.66] | [-1.04, 4.76] |
| Grip strength dominant hand | 0.7 | 3.95 | 3.87*** | -0.65 | -0.79 | -0.01 |
|  | [-1.10, 2.50] | [-0.27, 8.17] | [1.63, 6.10] | [-2.25, 0.95] | [-2.76, 1.19] | [-2.29, 2.26] |
| ***Health behaviours*** |  |  |  |  |  |  |
| Problematic alcohol consumption (OR) | 0.78 | 0.71 | 1.33 | 1.31 | 1.12 | 1.11 |
|  | [0.43, 1.40] | [0.40, 1.28] | [0.68, 2.61] | [0.58, 2.97] | [0.57, 2.20] | [0.44, 2.82] |
| Smoking current (OR) | 1.59 | 2.20* | 4.70*** | 1.19 | 2.32* | 2.53* |
|  | [0.53, 4.79] | [1.10, 4.40] | [2.47, 8.97] | [0.58, 2.43] | [1.17, 4.62] | [1.19, 5.38] |
| Daily step counts | 23.93 | -1247.35 | -1445.26 | -618.99 | -243.64 | -1000.46 |
|  | [-826.29, 874.16] | [-2775.37, 280.68] | [-3694.54, 804.01] | [-1550.27, 312.28] | [-1801.93, 1314.65] | [-2749.40, 748.48] |
| Mean activity (minutes/day) | -0.32 | -9.86 | -11.06 | -1.33 | -1.09 | -6.79 |
|  | [-6.21, 5.56] | [-20.63, 0.91] | [-23.42, 1.30] | [-8.36, 5.70] | [-11.42, 9.23] | [-18.20, 4.61] |

*Notes:* [. ] are 95% confidence intervals. * p<0.05, ** p<0.01, *** p<0.001

^a^ Odds ratios from an ordered logit model

^b^ Among those who are employed

CL = Childhood limited, AO = Adolescent onset, EOP = Early onset persistent. IRR = Incidence Rate Ratio

IPWs are used in all regressions.

1. Plewis I, Calderwood L, Hawkes D, & Nathan G. (2004). *Changes in the NCDS and BCS70 populations and samples over time*. CLS Technical Report, London: Centre for Longitudinal Studies. [↑](#footnote-ref-1)
